# Effect of external isometric hip rotation force on lower extremity muscles activities during pelvic drop with different hip positions

**DOI:** 10.1038/s41598-022-26472-9

**Published:** 2022-12-19

**Authors:** Roghayeh Jalil piran, Farideh Babakhani, Ramin Balouchi, Mohamadreza Hatefi

**Affiliations:** 1 Department of Sports Injury and Corrective Exercise, Faculty of Physical Education, Allameh Tabataba’i University, Tehran, Iran; 2 Department of Biomechanics and Sport Injuries, Faculty of Physical Education, Kharazmi University, Tehran, Iran

**Keywords:** Anatomy, Health care

## Abstract

Gluteus medius muscle (Gmed) dysfunction has been confirmed as a functional defect in subjects with Genu Valgum Deformity (GVD). The purpose of this study was to determine whether the change in the positions of hip rotation and applying isometric hip external rotation during pelvic drop (PD) can affect muscles activity in subjects with GVD. A total of thirty recreational female athletes with (n = 15) and without (n = 15) GVD participated in this study. Surface electromyography measured Gmed, tensor fascia latae (TFL), and quadratus lumborum (QL) muscles activity when subjects performed PD in three different positions of hip rotations with and without applied isometric hip external rotation force. There were differences in muscle activity between GVD and healthy subjects. The Gmed/TFL and Gmed/QL muscles activity ratio altered while placing the hip in different rotation positions and applying isometric load. The lower extremity muscles’ activity is affected by GVD, and changing the positions of the hip rotation in the PD task can be associated with altered muscle activity in both GVD and healthy Groups. However, applying isometric hip external rotation during PD can be suggested as an effective intervention to increase Gmed activity.

## Introduction

Genu valgum deformity (GVD) is defined as a set of lower extremity kinematic changes, including increased hip adduction and internal rotation of the proximal components, knee abduction, and external rotation of the distal components^1,2^, which is more common in women than men due to anatomical differences, particularly in quadriceps angle^3^. Given the functional activities begin from the static positions, it is clear that individuals with GVD are accompanied by movement patterns dysfunctions such as excessive hip adduction and internal rotation during various activities^4–6^, which has been described as a risk factor for acute (Including ACL injury) and chronic lower extremity injuries such as patellofemoral pain syndrome (PFPS) and iliotibial band syndrome (ITBS)^7,8^.

In relation to subjects with GVD, several studies have recommended that the impaired movement patterns could be improved by strengthening the gluteus medius (Gmed) muscle as a hip adduction and internal rotation controller^9–11^; It has been introduced as a major mechanism in controlling knee dynamic valgus. For example, it is shown that impairment in the production of hip abductors strength can increase the range of motion of hip adduction and internal rotation during weight-bearing activities, this potentially affects the kinematics of the lower extremities and causes knee valgus^12^. In fact, there is a correlation between hip muscle strength and lower extremity kinematics. In this regard, there is a theory that strengthening the muscles in isolation cannot be associated with the correction of movement patterns, but the correction of the movement patterns during functional activity will be accompanied by strengthening of muscles in the appropriate length and intensity, and changes in the neuromuscular system^2^. Therefore, it is important to design training protocols in functional conditions to change hip neuromuscular control aiming to affect the lower extremity kinematics.

On the other hand, the synergist muscles work together and affect each other^13–16^, therefore it is important to examine them together in electromyography studies. Because it is shown that muscle imbalance or changes in muscle activity patterns between them are associated with non-contact injuries such as PFPS and ITBS^7^. According to Selkowitz et al.^15^ increased TFL/Gmed activity ratio can cause patellar lateral tracking via increasing the external force by connecting the iliotibial band to the patella, which is associated with PFPS and ITBS. Moreover, insufficiency in the Gmed performance will be associated with increased opposite side quadratus lumborum (QL) activation as a synergistic muscle to prevent pelvic drop, which is causing QL over-activity and trigger points^17,18^. In this regard, clinical experts often report that an imbalance between Gmed and QL or a decrease in Gmed/QL activity ratio can induce movement impairment and low back pain^19^. In a similar study of isometric hip external rotation during single-leg stance, Schmitz et al.^20^ detected an increase in Gmed activity in response to isometric hip external rotation force. On the other hand, changes in the positions of hip rotations have been shown to be associated with changes in Gmed activity and can cause altered lower extremity alignment or be itself affected by postural malalignment^13,21^.

Therefore, the purpose of this study was to determine whether the change in the positions of hip rotation and applying isometric hip external rotation during PD can affect muscles activity in subjects with GVD. The main hypothesis of our research was that there is a difference between the muscle activity of subjects with and without GVD during the PD task. Also, according to previous studies, a change in the hip rotation positions be associated with a change in muscle activity, and we also had the same idea. Ultimately our hypothesis was that the Gmed/TFL activity ratio increases after isometric hip external rotation intervention during PD.

## Methods

### Participants

This study was designed as a controlled laboratory study with a pre-post intervention trial. According to G. Power software, with a power of 0.95, an effect size of 0.45, and an alpha level of 0.05, 30 recreational female athletes in age 18 to 25 years were selected in this study (G*Power, Franz Faul University of Kiel, Germany), which were divided into two groups: healthy (n = 15) and GVD (n = 15). The GVD was defined as the distance between the two medial malleolus greater than 5 cm in the standing position; evaluation by a corrective exercise specialist^22–24^. Participants were invited to the present study through the board of the university and local sporting clubs. Inclusion criteria were: exercise at least three times a week and continuously, age between 18–25 years, and body mass index (BMI) between 18 and 24. Participants were excluded if they: had abnormalities of the pelvic and lumbar regions including hyperlordosis or any osteogenic deformities, had any musculoskeletal injury in the previous two months or lower-extremity injury in the previous six months, had a lower limb surgery or fractures within the past one year, had any neurological and pathological conditions, or inability to perform the exercise due to lack of understanding of the relevant movement. Also, all subjects provided written informed consent prior to participation in the study. Ethical approval was granted by the ethical committee of the Allameh Tabataba’i University ethic board (IR.ATU.REC.1399.016). Also, we confirm that all methods were performed in accordance with the relevant guidelines and regulations.

### Procedures

**Figure 1:**
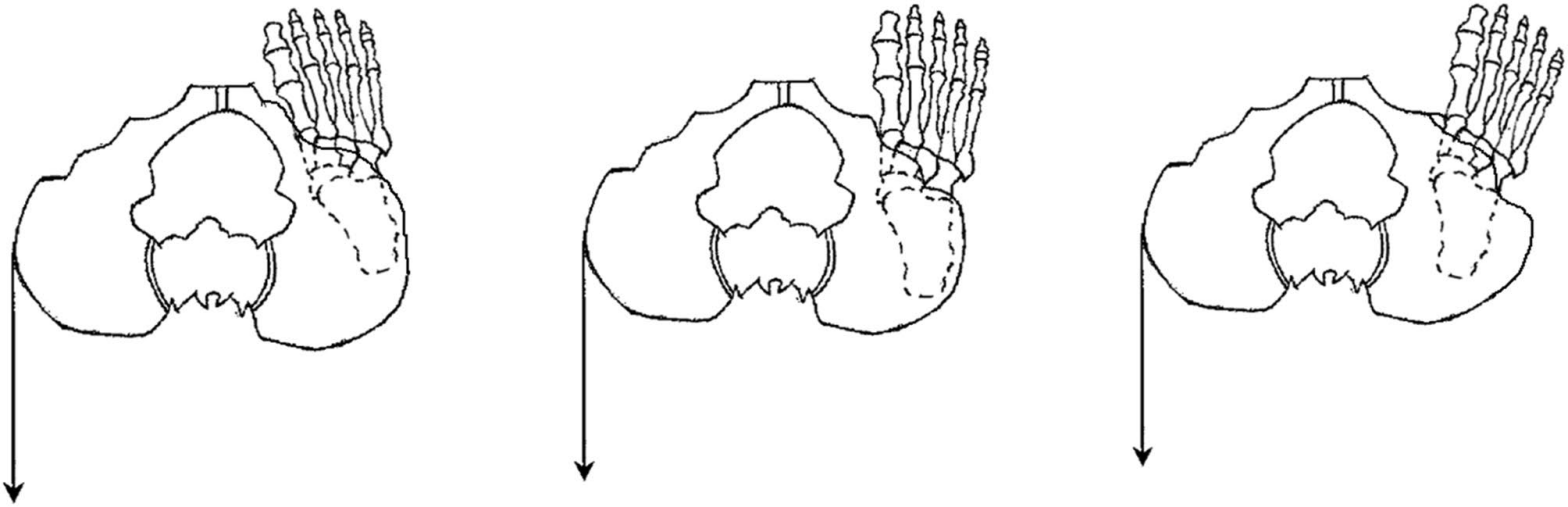
Superior-to-inferior view of performing PD in different positions of hip rotation by applying isometric force to the outer edge of the pelvic of the non-stance side (isometric hip external rotation of stance side).

In the present study, participants were referred to the laboratory once/ one time and completed a 1-h test session. They were asked to wear comfortable sports clothing without shoes aiming to prevent the influence of footwear differences. In general, Gmed, TFL, and QL activity were recorded while participants performing PD in three different positions of hip rotation (neutral, 15° internal, 20° external rotation) with and without isometric external load. In order to prevent possible fatigue, they were given rest for one minute between different positions of hip rotation and 4 min between different load conditions. It is noteworthy that prior to performing the PD, participants pedaled with a cycle ergometer at a self-selected speed and low resistance for 5 min as a warm-up. To determine the different positions of hip rotation, the plate was used to adjust these positions, and the neutral, 15° internal rotation (IR), and 20° external rotation (ER) were drawn (Fig. 1). Then, the participants stood on the plate in a position so that the middle line passed posteriorly through the central calcaneus and anteriorly through the second toe. For hip rotation, the participants’ feet were placed on the angled lines as described above. Also, the EMG data were collected in the Gmed and TFL muscles of the dominant limb (support limb) and QL muscles of the opposite side.

To perform the PD task, the participants were asked to stand on their dominant leg on a 15-cm step, and lower the non-dominant heel towards the floor while maintaining the extension of both knees, then return the leg back to the first position (Fig. 1). The step height was adjusted for each of the participants so that the knee was positioned approximately at 30° flexion when the heel of the opposite leg touched the floor. Also, they were asked to lightly touch their heel to the floor aiming to ensure standardization and consistency between them and to ensure that adequate depth of the exercise was achieved each time.

To apply the isometric hip external rotation during PD, a tensiometer cable connected to a dynamometer was used while subject trying to perform task without disturbing their balance. In term of location and amount of the force, a load of 15 N in the back direction (posteriorly direction force) was applied to the outer part of the non-stance side of the pelvic (Fig. 1). The amount of force applied is determined using a pilot test, such that the balance of individuals is not disturbed and does not change the standing position. Participants are given verbal feedback during the process to prevent compensatory movement patterns such as trunk lateral flexion and rotation or pelvic rotation; In this case, the test was considered correct^20^. Also, a bar was placed horizontally in front of the body so that the bar passes over both the anterior superior iliac. Participants were asked not to move their bodies away from the bar during the test.Please note we have moved the section “Ethical approval and informed consent” to the end of the methods, as per house style.

### Muscles activity

Surface wireless EMG (Myon m320RX, Schwarzenberg, Switzerland) was used to quantify the TFL, Gmed, and QL activation. Raw EMG signals were recorded at the sampling frequency of 1000 Hz, full-wave rectified, and then data noise was filtered at the 20–490 Hz band-pass and smoothed by the symmetrical moving RMS filter. Muscles activity analyses were conducted for the pre and post-intervention during PD task in different positions of hip rotation. Notably, the beginning and end of the PD movement task were calculated based on the non-weight-bearing heel marker displacement. The EMG data were normalized to the maximal voluntary isometric contractions (MVIC). The mean activation for each muscle was divided by the corresponding MVIC, and the average EMG data during PD were expressed as a percentage of the MVIC. Eventually, the average of the 2 sets was used for statistical analysis. Also, all EMG data were processed using Matlab software (Mathworks, Natick, MA).

Based on SENIAM recommendation^25^, the electrodes were placed in the direction of muscle fibers and on the dominant leg, which was defined as the preferred one for kicking a soccer ball^26^. Before placing the electrodes, the skin surface was shaved, abraded, and cleaned with 75% alcohol to reduce skin resistance. MVIC were used as a standard protocol to normalize the EMG data. To perform MVIC, the standard manual muscle-test was used in specific positions for each muscle^1^. The MVIC of each muscle were performed for three trials of five seconds with one minute of rest between repetitions. To measure the MVIC for the TFL, subjects lying on their side and the lower extremity were placed at 45° hip flexion and 30° hip abduction, and the knee was extended. A leather or nylon band was tied around the ankle, individuals were asked to apply a force diagonally between the sagittal and frontal plane at an angle of approximately 45°. To measure the MVIC for the Gmed, the subjects were lying on his side, so that the dominant leg was upwards and the whole body was in one direction. The dominant hip was located without abduction/adduction and internal/external rotation. To better perform the move, it is best if the leg below is flexed. The investigator applied a downward force to the ankle, while the subjects were asked to resist the applied force isometrically^27^. To measure the MVIC for the QL, the subject lying on the side with an extension in the knee and places her hands on the opposite shoulder. A nylon or leather strap was tied around the ankle to prevent abduction and by placing a resistor on the person's shoulder, the subjects were asked to give lateral flexion to the trunk. For all muscles, verbal stimuli were given to maximize the motivational effect of subjects ^28^. The highest mean peak value of these three repetitions was recorded as the MVIC of each muscle from the relevant muscle manual test. Also, to calculate the activity ratio of Gmed / TFL and Gmed / QL muscles, the normalized mean EMG of the Gmed was divided by the normalized mean of the EMG of the TFL, and the normalized mean EMG of the Gmed was divided by the normalized mean of the EMG of the QL respectively. A ratio greater than 1 indicates that the Gmed activity was greater than that of the TFL and QL activity.

### Statistical analysis

Descriptive analysis (mean ± SD) was performed on all the variables. Given the normality of the data distribution based on the Shapiro–Wilk test, the one-way repeated measures ANOVA followed by post hoc Bonferroni test was used to compare the muscle activities in different positions of the hip rotation for each group separately. A paired t-test was used to compare the pre-post load intervention in each angle. Also, an independent t-test statistical test was used to compare the differences between the healthy and GVD groups.

Statistical analyses were performed using SPSS software Version 22 (Microsoft Corp., Redmond, WA) at the significant level of *p* ≤ 0.05.

### Ethical approval and consent to participate

All experimental protocols of this study were carried out following Declaration Helsinki and were approved by the Ethics Committee of Allameh Tabataba’i University (IR.ATU.REC.1399.016). All subjects provided written information consent prior to participation in the study.

## Results

Altogether 30 female recreational athletes (15 Healthy and 15 GVD) were included in the study. There was no significant difference in demographic characteristics between the two groups of healthy and GVD (p˃0/05), but the distance between the medial malleolus of the ankle between the two groups was statistically significant (p = 0.001) (Table 1).

**Table 1:** Demographic characteristics of the participants (Mean ± SD).

variable	Healthy (n = 15)	GVD (n = 15)	*p *value
Age (year)	21.33 ± 1.78	21.73 ± 2.12	0.589
Height(cm)	164.33 ± 1.91	164.4 ± 2.16	0.931
Weight (kg)	57.47 ± 1.64	58.2 ± 2.05	0.389
BMI(kg/m^2^)	21.29 ± 0.91	21.5 ± 1.22	0.588
Medial malleolus distance (cm)	1.37 ± 0.53	7.66 ± 0.83	0.001*

*Significant in *p* ≤ 0.05 level.

*GVD* Genu valgum deformity.

### Gmed, TFL, and QL activity

The results of the present study showed a decrease in Gmed activity in the 20°ER than neutral (with= 0.001, without=0.015) and 15°IR (with= 0.025, without=0.001) of hip position in both with and without load condition, increase in QL activity in 15°IR than neutral of hip position without load condition (*p*=0.008), and increase in QL activity in the 20°ER than neutral (with= 0.001, without= 0.001) and 15°IR (with= 0.001, without= 0.036) of hip position in both with and without load condition in the healthy group. In relation to GVD group, we showed a decrease in Gmed activity in the 20°ER than neutral (with= 0.001, without= 0.045) and 15°IR (with= 0.001, without= 0.005) of hip position in both with and without load condition, increase in QL activity in 15°IR than neutral of hip position without load condition (*p*= 0.025), and increase in QL activity in the 20°ER than neutral (*p*=0.001) and 15°IR (*p*=0.015) of hip position in load condition.

The results based on the independent t-test showed a significant difference in Gmed activity at 15°IR and neutral of hip position, TFL activity at all three positions of hip rotation in both with and without load condition between the two groups. On the other hand, the results showed that there was a significant difference in the QL activity at 20 ° ER and neutral of hip position in both with and without load conditions between two groups. However, a significant difference in 15° IR of hip position only was seen in load condition (*p*<0.05) (Table2).

**Table 2:** Mean ± Standard deviation of normalized RMS EMG data for each muscle during the pelvic drop in different positions of hip rotation with and without load (isometric hip external rotation).

Variables		Neutral		15° IR		20° ER		Repeated measures ANOVA, *p*-value	
Muscles (%MVIC)	Group	No load	Load	No load	Load	No load	Load	No load	Load
Gmed	Healthy	84.88 ± 15.03^#a^	87.70 ± 28.40^a^	87. 6±14.61^#a^	90.81 ± 23.5^a^	42.61 ± 11.12^bc^	42.63 ± 13.15^bc^	0.001*	0.001*
	GVD	42.73 ± 12.05^a^	43.75 ± 10.02^ab^	42.67±10.01^#a^	45.78 ± 9.01^ac^	40.67 ± 8.01^bc^	38.66 ± 7.25^bc^	0.001*	0.001*
	Independent t-test, *p*-value	0.001*	0.001*	0.001*	0.001*	0.344	0.400		
TFL	Healthy	70.76 ± 21.02^#ab^	72.82 ± 15.04^b^	70.75±16. 02^#ac^	68.55 ± 14.22^ac^	70.82 ± 13.04^bc^	70.82 ± 18.04^b^	0.001*	0.001*
	GVD	70.88 ± 18.08^ab^	70.88 ± 17.24	70.85±15.16^#c^	73.88 ± 17.24	70.86 ± 16.22^#c^	16.17 ± 68.78	0.005*	0.215
	Independent- t-test, *p*-value	0.085	0.152	0.468	0.168	0.525	0.157		
QL	Healthy	56.68 ± 13.04^ab^	55.66 ± 12.02^a^	58.76±11.05^#ac^	54.65 ± 14.09^a^	60.17 ± 15.45^#bc^	62.22 ± 14.53^bc^	0.001*	0.001*
	GVD	56.76 ± 14.02^#a^	59.84 ± 14.11^ab^	57.74±14.01^#a^	54.74 ± 13.21^c^	57.25 ± 16.58^#ab^	53.76 ± 17.05^c^	0.001*	0.001*
	Independent- t-test, *p*-value	0.001*	0.001*	0.378	0.001*	0.003*	0.001*		

*IR* Internal rotation of hip position, *ER* External rotation of hip position.

*p ≤ 0/05: Bonferroni post hoc test: ^a^a significant difference with 20^0^ external rotations, ^b^a significant difference with 15^0^ internal rotation, ^c^a significant difference with neutral. ^#^a significant difference between pre-and post-load intervention based on the paired t-test.

### Gmed/TFL and Gmed/QL activity ratios

In relation to the healthy group, the results showed a decrease in Gmed/TFL activity ratio in the 20°ER than neutral (with= 0.006, without=0.001) and 15°IR (with= 0.001, without=0.035) of hip position in both with and without load condition, decrease in Gmed/QL activity ratio in 20°IR than neutral (with= 0.001, without=0.001) and 15°IR (with= 0.007, without=0.045) of hip position in both without load condition. Also, we also observed an increase in Gmed/TFL activity ratio at 15°IR of hip position after load intervention (*p*= 0.001).

In relation to the GVD group, we showed an increase in Gmed/TFL activity ratio in the 20°ER than neutral (*p*=0.012) and 15°IR (*p*=0.001) of hip position in without load condition, increase in Gmed/QL activity ratio in 20°IR than 15°IR (*p*=0.014) of hip position in without load condition, and increase in Gmed/QL activity ratio in 20°IR than neutral (*p*=0.048) and 15°IR (*p*=0.001) of hip position in with load condition. We also observed an increase in the Gmed/QL activity ratio at all three positions of hip rotation after load intervention (*p*<0.05).

The results based on the independent t-test showed a significant difference in Gmed/TFL activity ratio at 15°IR and neutral positions of hip rotation, the Gmed/QL activity ratio at all three positions of hip rotation in both with and without load condition between the two groups. (Table 3).

**Table 3:** Mean ± Standard deviation of muscles activity ratio during the pelvic drop in different positions of hip rotation with and without load (isometric hip external rotation).

Variables		Neutral		15° IR		20° ER		Repeated measures ANOVA, *p*-value	
Muscles activity ratio	Group	No load	Load	No load	Load	No load	Load	No load	Load
Gmed/TFL ratio	Healthy	1.19 ± 0.55^a^	1.24 ± 0.40^a^	1.23 ± 0.31^#a^	1.28 ± 0.33^a^	0.99 ± 0.21 ^#cb^	0.90±0.19^cb^	0.018*	0.001*
	GVD	0.60 ± 0.17^ba^	0.60 ± 0.11	0.60 ± 0.12^ac^	0.60 ± 0.11	1.0 ± 0.15 ^#cb^	0.60±0.16	0.001*	0.001*
	Independent t-test, *p*-value	0.001*	0.001*	0.001*	0.001*	0.990	0.990		
Gmed/QL ratio	Healthy	1.49 ± 0.31^a^	1.54 ± 0.50^a^	1.54 ± 0.39^a^	1.6 ± 0.41^a^	0.73 ± 0.21^cb^	0.73±0.16^cb^	0.001*	0.001*
	GVD	0.73 ± 0.13^#^	0.75 ± 0.14b^a^	0.72 ± 0.13^#a^	0.78 ± 0.14^ac^	0.74 ± 0.11^#b^	0.79±0.12^cb^	0.001*	0.001*
	Independent- t-test, *p*-value	0.001*	0.001*	0.001*	0.001*	0.014*	0.001*		

*IR* Internal rotation of hip position; *ER *External rotation of hip position.

*p ≤ 0/05: Bonferroni post hoc test: ^a^a significant difference with 20^0^ external rotations, ^b^a significant difference with 15^0^ internal rotation, ^c^a significant difference with neutral. ^#^a significant difference between pre-and post-load intervention based on the paired t-test.

## Discussion

The main purpose of our study was to determine muscle activity differences between the healthy and GVD subjects during the PD task. In the second purpose, we wanted to know if placing the hip in different rotation positions or applying isometric external load during the PD task increases Gmed and Gmed/TFL ratio activity. As hypothesized, the results of this study indicated a statistically significant difference in muscles activity between healthy and GVD subjects. In general, we observed a decrease in Gmed activity and an increase in TFL and QL activity in different conditions in GVD compared to healthy subjects. However, the muscles activity ratio was altered with a change in hip rotation positions and applying external force during the PD task.

Considering the importance of strengthening the Gmed muscle, as a basic strategy especially in subjects with GVD, many studies have investigated the effect of various interventions on increasing Gmed activity aiming to improve hip adduction and internal rotation control as well prevent non-contact injuries such as ITBS and PFPS injuries^14,15,21,29–32^. In this regard, Distefano et al.^33^ reported that rehabilitation and prevention of lower extremity injuries often include exercises that are performed at different levels of difficulty one of the main goals is to increase gluteal muscle activity. Notably, when designing an exercise to increase the activity of a specific muscle, we have to consider its synergist muscles as well. Synergist muscles work together and affect each other during movement^14,15,34^. In our study, understanding the muscles activity ratio during the PD task with different load and hip position conditions may provide clinical information as to which hip rotation positions preferentially activate the Gmed, while minimizing the QL and TFL activity. Because synergist muscle dominance has been shown to impair motor control and ultimately cause non-contact injuries^34^. For example, it has been shown that Gmed activity has a negative correlation in relation to the QL activity during functional activities; which can lead to the impaired motor control as well as low back pain^19,34^.

The results of the current study in relation to healthy subjects replicated the findings of the previous study; which found statistically significant altered muscles activity during the PD task in different positions of hip rotation; which showed an increase in Gmed activity and Gmed/TFL activity ratio during the PD task with the medial position of hip rotation compared to the other position^18,21^. This finding may be explained by the fact that placing the hip in the medial position of hip rotation may increase the Gmed muscle's rest length; which would produce greater activity by altering the position of the sarcomeres. In contrast, we observed a decrease in Gmed activity and Gmed/TFL activity ratio in the 20°ER position of the hip rotation due to Gmed active insufficiency. This concept implies that muscle shortening leads to low tension. Also, we observed an increase in Gmed activity at neutral and 15°IR of the hip position after applying external force. However, this increased activity did not change the muscle activity ratio as well as the difference between hip rotation positions.

On the other hand, our results indicate that GVD modifies the Gmed function during PD. The results of the current study indicate a decrease in Gmed activity in all hip rotation positions compared to the healthy group. In other words, we observed increased TFL and QL activity as a result of reducing Gmed activity to complete movement and maintain pelvic stability during the PD task. The results presented in this section support the theory of the relationship between weakness in a muscle and dominance of the synergist muscles as the primary muscle^15^. However, the applying isometric hip external rotation load was associated with increased Gmed activity in these subjects. Exactly, we observed an increase in Gmed/TFL and Gmed/QL activity ratio after applying external load intervention in the neutral and 15°IR of hip position. Notably, due to dysfunction in the hip adduction and internal rotation control during various activities in subjects with GVD, we do not recommend performing PD exercise in the 15°IR of hip position. Although it is confirmed that the muscles should be strengthened in their optimal joint position especially in subjects with postural malalignment, otherwise it changes the movement pattern over time. Thus, consistent with recommended clinical practice, performing the PD task in the neutral hip position by applying the external load can be considered as an effective exercise to improve the Gmed and Gmed/TFL activity ratio.

Nevertheless, this study has several limitations. First, this is a cross-sectional study, so its long-term effects are unclear. Second, the neutral hip position angle is subjective, however, we did not consider that in this study. Third, the subjects were female recreational athletes, so these results may not be generalizable to everyone. Furthermore, gender differences may play an important role in muscle activity patterns. And finally, the small sample size could be considered as another current study limitation.

## Conclusion

The lower extremity muscles activity is affected by GVD, and changing the positions of the hip rotation in the PD task can be associated with altered muscle activity in both GVD and healthy Groups. However, applying isometric hip external rotation during PD can be suggested as an effective intervention to increase Gmed activity.

## Data Availability

The datasets generated during and/or analysed during the current study are available from the corresponding author on reasonable request.

## Abbreviations

GVD: Genu valgum deformity
PD: Pelvic drop
Gmed: Gluteus medius
TFL: Tensor fascia latae
QL: Quadratus lumborum
PFPS: Patellofemoral pain syndrome
ITBS: Iliotibial band syndrome
